# Facing environmental predictability with different sources of epigenetic variation

**DOI:** 10.1002/ece3.2283

**Published:** 2016-06-28

**Authors:** Christelle Leung, Sophie Breton, Bernard Angers

**Affiliations:** ^1^ Department of Biological SciencesUniversité de MontréalC.P. 6128, succ. Centre‐villeMontrealQuebecH3C 3J7Canada

**Keywords:** Bet‐hedging, *Chrosomus eos‐neogaeus*, clonal organisms, environmental variation, epigenetics, phenotypic plasticity

## Abstract

Different sources of epigenetic changes can increase the range of phenotypic options. Environmentally induced epigenetic changes and stochastic epimutations are, respectively, associated with phenotypic plasticity and diversifying bet‐hedging. Their relative contribution is thus expected to reflect the capacity of a genotype to face distinct changes since these strategies are differentially selected according to environmental uncertainty. To test this hypothesis, we assessed the sources of epigenetic changes on clonal fish from predictable (lakes) or unpredictable (intermittent streams) environments. DNA methylation of clones from natural conditions revealed contrasting contribution of environmentally induced versus stochastic changes according to their origins. These differences were validated in common garden experiments. Consistent with theoretical models, distinct sources of epigenetic variation prevail according to the environmental uncertainty. However, both sources act conjointly, suggesting that plasticity and random processes are complementary strategies. This represents a rigorous approach for further exploring the capacity of organisms to respond to environmental conditions.

## Introduction

Epigenetic processes are known to extend the phenotypic options of a genotype by fine‐tuning gene expression and triggering development of alternative phenotypes (Jaenisch and Bird 2003; Kucharski et al. 2008; Matsumoto et al. 2013). Environmentally induced and spontaneous stochastic modifications (epimutations) are two fundamentally different mechanisms enabling epigenetic variation. Following the perception of an environmental signal, specific genes may be epigenetically silenced or activated, resulting in a modified and environment‐specific phenotype (Rando and Verstrepen 2007; Chinnusamy and Zhu 2009; Beldade et al. 2011; Verhoeven and Preite 2014). Environmentally induced epigenetic variation has therefore been proposed to mediate phenotypic plasticity (Angers et al. 2010; Bollati and Baccarelli 2010). This strategy depends on the perception and integration of environmental signals during development (Pigllucci 1996; Bateson et al. 2014) and thus results from interactions between environmental stimuli and epigenetic processes. Theoretical studies demonstrated that phenotypic plasticity would be selected for dealing with predictable environmental changes, for example, when environmental conditions change between generations but remain stable within a generation, allowing organisms to develop proper phenotypes until reproduction (Lachmann and Jablonka 1996; DeWitt et al. 1998; Reed et al. 2010; Scheiner and Holt 2012).

On the other hand, stochastic epigenetic changes can also result in the production of different phenotypes (Cubas et al. 1999; Rakyan et al. 2002; Manning et al. 2006; Miura et al. 2009). Stochastic variation may be generated because of the high error rate of DNA methyltransferase in the establishment of methylation marks during DNA replication (Riggs et al. 1998). The rate of epigenetic changes is extremely variable, and epimutations can be 10^4^ times higher than somatic mutations in some genes (Bennett‐Baker et al. 2003; Massicotte et al. 2011; Schmitz et al. 2011). Epimutations can occur spontaneously (Becker et al. 2011), but their rates may also increase when organisms are exposed to environmental stresses (Rapp and Wendel 2005). The stochastic establishment of epigenetic marks has been proposed to be among the potential mechanisms underlying diversified bet‐hedging strategy (Piggot 2010; Casadesús and Low 2013; Herman et al. 2014; Vogt 2015). Indeed, this risk‐spreading strategy is based on the stochastic production of phenotypically variable offspring, irrespective of environmental conditions (Slatkin 1974; Veening et al. 2008; de Jong et al. 2011). Stochastic production of phenotypes makes this strategy more advantageous to organisms coping with unpredictable environmental changes, for example, when environmental signals experienced during development do not predict environmental conditions that individuals will cope with until reproduction (Balaban et al. 2004; Kussell and Leibler 2005; Acar et al. 2008; Fraser and Kærn 2009; Rajon et al. 2014; Scheiner 2014; Botero et al. 2015).

Either environmentally induced or stochastic DNA methylation variation can alter gene expression (Wolff et al. 1998; Morgan et al. 1999; Kucharski et al. 2008). These mechanisms are not alternative to each other but complementary in producing phenotypic diversity in the absence of genetic variation. The relative contribution of environmentally induced and stochastic epigenetic variation is thus expected to differ according to the predictability of environmental changes and reflect the capacity of a given genotype to be either more plastic or more bet‐hedger. However, this hypothesis, fundamental to our understanding of the capacity of a given genotype to respond to environmental conditions, still needs to be empirically tested.

Our study aims at determining whether distinct contribution of environmentally induced epigenetic changes versus randomly established epigenetic marks would be observed according to the predictability of environmental changes. Theoretical models predict that organisms found in predictably or unpredictably changing environments are, respectively, more likely to be plastic or adopt bet‐hedging strategy (Kussell and Leibler 2005; Scheiner and Holt 2012; Starrfelt and Kokko 2012; Scheiner 2013). Therefore, our prediction is that high proportion of environmentally induced epigenetic changes will be present in organisms distributed in multiple and spatially different but predictable environments, whereas high randomly established epigenetic marks will be present in organisms living in unpredictable environments.

**Figure 1:**
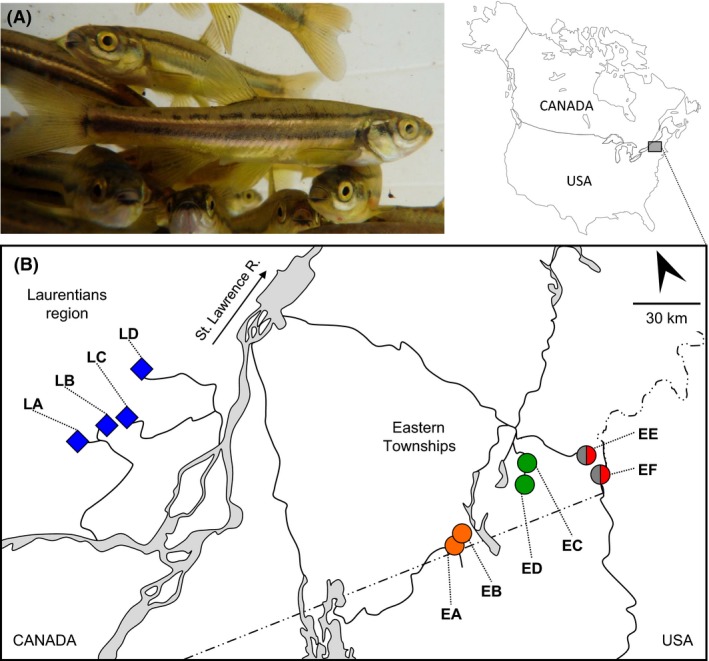
Model species *Chrosomus eos‐neogaeus* and study sites. (A) Adult individuals of the complex *C. eos‐neogaeus*. (B) Map of southern Quebec (Canada) indicating the field sites in Eastern Townships (circles) and Laurentians (diamonds) regions. Colors refer to distinct hybrid lineages (LR‐1 = blue, ET‐1 = green, ET‐2 = orange, ET‐3 = gray and ET‐4 = red) and site names are in capital letters.

We assess environmentally induced and stochastic epigenetic variation on a clonal organism to rule out the effect of DNA polymorphism on epigenetic variation by analyzing replicates of a genotype within and among environments. Specifically, the asexual *Chrosomus eos‐neogaeus* hybrid fish (Fig. 1A) provide a suitable system as several lineages from distinct hybridization events occur naturally (Schlosser et al. 1998; Angers and Schlosser 2007). These fish are found in different and contrasting environmental conditions such as ponds, lakes, and ephemeral streams (Scott and Crossman 1973; Goddard et al. 1998; Schlosser et al. 1998), which present different level of environmental predictability according to the fish ecology (Giller and Malmqvist 1998; Wetzel 2001). Lakes are considered as stable and predictable environments compared to intermittent headwater streams given the morphology and the short‐term variability in physicochemical conditions of the latter (Wetzel 2001). Intermittent headwater streams present more unstable channel morphology because of flowing water erosive action, resulting in a higher sensitivity to flood variation and a higher dynamism and unpredictability in fish habitats. Moreover, streams respond more rapidly to precipitation events, resulting in faster and larger changes in both physical and chemical conditions (Weyhenmeyer et al. 2012). Because the physicochemical conditions of water are primary regulators of species composition, the numbers/diversity of prey as well as predators is therefore more unpredictable in intermittent streams than in lakes and ponds.

Environmentally induced and spontaneous stochastic epigenetic changes were assessed by partitioning of epigenetic variability within clonal lineages from distinct natural populations of *C. eos‐neogaeus*. Specifically, environmentally induced variation was inferred from epigenetic differences among sampling sites while stochastic changes referred to residual epigenetic variation, explained neither by genetic variation nor sampling sites. Because confounding effects may be encountered in the epigenetic signals from natural populations, epigenetic variation was also analyzed in individuals reared in common garden experiments.

## Materials and Methods

### Ethics statement

This research was performed under institutional animal care guidelines (permit #13‐084 delivered by the *Université de Montréal*) and conforms to the mandatory guidelines of the Canadian Council on Animal Care. Sampling permits were provided by the Quebec Ministry of Natural Resources and Wildlife (MRNF).

### Biological model, sampling and genetic identification

The asexual *C. eos‐neogaeus* results from hybridization events between the redbelly dace *C. eos* and the fine‐scale dace *C. neogaeus* (Dawley et al. 1987) and reproduces clonally by gynogenesis (Goddard and Dawley 1990; Goddard et al. 1998). Distinct hybridization events have resulted in the formation of multiple lineages (Angers and Schlosser 2007). Hybrids are present in contrasting environments (Schlosser et al. 1998). In addition, both environmental and stochastic effects on DNA methylation have been previously reported as sources of epigenetic variation in *C. eos‐neogaeus* (Massicotte et al. 2011; Massicotte and Angers 2012).

Two regions in southern Quebec (Canada) where the presence of different *C. eos‐neogaeus* lineages has been reported (Angers and Schlosser 2007) were retained for this study. Sampling of adults *C. eos‐neogaeus* was conducted in lakes (predictable environments) of the Laurentians region (LR‐1 lineage) and in Eastern Townships (ET‐2 to ET‐5 lineages) streams (unpredictable environments) (Fig. 1B).

Total DNA from the caudal fin of each individual was extracted according to Sambrook et al. (1989). Genetic identification was performed according to Binet and Angers (2005). The assignment of individuals to a given lineage was achieved using the multilocus genotype of eight hypervariable microsatellites amplified according to Angers and Schlosser (2007): Pho‐1, Pho‐2, Pho‐60, and Pho‐61 specific to *C. eos* and Ca‐12, Seat‐412, amplifying both *C. eos* and *C. neogaeus* haplomes (Binet and Angers 2005; Skalski and Grose 2006; Angers and Schlosser 2007). A lineage is defined as all individuals expected to originate from a given hybrid zygote; in the absence of mutation, recombination, and segregation, individuals of a given lineage are thus expected to be genetically identical (Angers and Schlosser 2007). Given the high mutation rate of microsatellite loci, mutations (generally one or a few number of repeats away from the consensus size) can be observed at one or few loci between individuals of a given lineage (Angers and Schlosser 2007). Therefore, these markers allow the assessment of genetic differences among individuals within a given lineage.

### Common garden experiments

The common garden experiments allowed the elimination of most of the confounding factors (e.g., lineages found in different environmental conditions or genetic variation among individuals from distinct sites). The experimental setup was designed to provide a stable and homogeneous environment in an aquarium: natural photoperiod, constant temperature at 19°C, saturated oxygenation, and ad libitum feeding to avoid competition. Thirty fish larvae (<1 cm) per lineage were sampled randomly from natural populations for lineages LR‐1 from the lake LD (46°05′35.5″N 73°52′15.7″W) and ET‐2 from the intermittent stream EA (45°02′35.8″N 72°21′43.1″W) (Fig. 1B; Table 1). Individuals were then reared together in a constant and uniform environment until adults. After 5 months in the experimental conditions, individuals reached approximately 5 cm; they were sacrificed and genetically identified according to the procedure described above.

**Table 1:** Epigenetic and genetic intrasite variation for each lineage. Individuals were grouped according to lineage and sampled environment. For each group, intra‐environment epigenetic variation (*E*‐_INTRA_) refers to mean individuals’ distance to centroid (Anderson 2006) with standard deviation (SD) and gene diversity was estimated with Nei's gene diversity (Nei 1978)

Lineages	Environments	Geographic coordinates		Sampling size	Epigenetic diversity		Gene diversity (*H*_E_)
					Number of variable loci	*E‐*_INTRA_ (*±*SD)	
LR‐1	LA	45°54′18.9″N	74°19′20.8″W	10	12	1.48 (±0.29)	0.07
	LB	45°55′03.1″N	74°04′30.0″W	30	7	1.21 (±0.14)	0.03
	LC	45°57′58.3″N	74°01′43.5″W	10	3	0.83 (±0.17)	0.14
	LD	46°05′32.4″N	73°52′09.6″W	8	3	0.90 (±0.11)	0.06
			Total	58	40		0.15
LR‐1	Common garden	–	–	10	2	0.57 (±0.32)	0.08
ET‐1	EC	45°14′01.8″N	71°54′28.0″W	10	22	2.08 (±0.15)	0.14
	ED	45°12′56.7″N	71°54′31.5″W	8	19	2.12 (±0.29)	0.00
			Total	18	27		0.10
ET‐2	EA	45°02′35.8″N	72°21′43.1″W	10	25	2.12 (±0.41)	0.21
	EB	45°03′01.4″N	72°19′03.2″W	10	22	2.04 (±0.41)	0.23
			Total	20	31		0.25
ET‐2	Common garden	–	–	10	9	1.37 (±0.23)	0.15
ET‐3	EE	45°11′04.5″N	71°33′13.2″W	29	12	1.28 (±0.30)	0.12
	EF	45°09′15.1″N	71°32′53.3″W	13	12	1.35 (±0.43)	0.15
			Total	42	16		0.15
ET‐4	EE	45°11′04.5″N	71°33′13.2″W	10	13	1.52 (±0.22)	0.29
	EF	45°09′15.1″N	71°32′53.3″W	8	10	1.33 (±0.38)	0.15
			Total	18	15		0.38

### Methylation‐sensitive amplified polymorphism analysis

Because epigenetic profiles are tissue specific (Shiota et al. 2002; Massicotte et al. 2011), the survey of DNA methylation was exclusively performed on the caudal fin. Epigenetic analysis was performed on 156 individuals from five lineages in natural populations (Table 1). An additional 20 individuals from two lineages (10 individuals randomly selected for LR‐1 and ET‐2) reared in controlled conditions were analyzed.

Survey of DNA methylation was performed using methylation‐sensitive amplified polymorphism (MSAP) technique (Xiong et al. 1999). This method allows for a genome‐wide analysis of methylation patterns by using a subsample of the whole variation. Massicotte et al. (2011) confirmed that loci targeted by MSAP method are functionally relevant, being located within genes, in gene regulatory regions or within transposable elements. In this study, only the frequent cutter *MspI* or *HpaII* restriction enzymes were used to increase the number of restriction sites within a selective PCR. Three selective PCR were performed using *MspI/HpaII*‐CAG, *HpaII*‐CCT, and *HpaII*‐CTC primers for both *MspI* and *HpaII* treatments. Loci were separated by electrophoresis on denaturing 6% polyacrylamide (19:1 acrylamide: bis‐acrylamide) gels and visualized by silver nitrate staining (Benbouza et al. 2006). Banding pattern was used to construct a presence/absence matrix for both treatments. Loci within the *HpaII* treatment associated with polymorphic loci within the *MspI* treatment were excluded since these specific bands are likely associated with genetic variation. To assess the reproducibility of the method, three separate MSAP reactions were performed for a subsample of 20 individuals randomly chosen. Only clearly amplified and reproducible bands over the three replicates were retained for analyses and nonreproducible loci were discarded for all individuals.

### Statistical analysis

The statistical programming environment R 3.2.2 was used for the computation of all statistics. Specifically, we used the *vegan* package 2.3‐2 (Oksanen et al. 2015) for multivariate analyses and *adegenet* 2.0‐0 (Jombart 2008; Jombart and Ahmed 2011) for genetic analyses.

Euclidean distances among individuals were computed from the presence/absence matrix of MSAP bands and were used to infer unrooted trees using the neighbor‐joining method. Relative support for the different groups was assessed by bootstrap analysis with 1000 replicates.

Partial redundancy analyses (Borcard et al. 1992) were used to determine the relative contribution of the lineage and the environment (different sampling sites or aquarium) on the total epigenetic variation. To investigate the extent to which epigenetic variation was affected by the environment for a given lineage, we performed redundancy analyses by taking into account the degree of genetic differences among individuals. First, genetic distances were calculated according to the number of mutation steps among individuals of a given lineage. Genetic distance was thereafter summarized according to a principal coordinate analysis. The percentages of the total epigenetic variation that can be attributed to the different factors (genetics and environment) were based on the adjusted *R* ^2^ ($R_{a}^{2}$) and significance of each fraction was tested by permutation tests using 999 randomizations (Peres‐Neto et al. 2006). This approach has been shown to be the most appropriate to decompose the variation according to different factors (Legendre et al. 2005) and provides a standard and comparable measure to describe the environmental effect for the different lineages.

Stochastic epigenetic variation was assessed with the residual epigenetic variation that cannot be attributed to either genetics or the environment. In natural populations, confounding effect (e.g., intrasite genetic composition and/or spatial heterogeneity) may, however, blur the environmental effect on epigenetic variation. Hence, stochastic epigenetic variation was also assessed according to the variation within a homogeneous environment (common garden) for two lineages compared to the variation within their respective natural populations. Variation was assessed according to the dispersion of individuals within a given environment, that is, mean distance to centroid (Anderson 2006). Analyses of multivariate homogeneity of group dispersions, a multivariate analogue of Levene's test, were performed using Euclidean distances matrix. Each group was defined by all individuals of a given lineage and from the same environment. Distances between individuals and group centroids were then computed with a bias correction for unequal sample sizes (Stier et al. 2013). Pairwise comparisons of mean distance to centroid of each lineage within a given environment were performed with 999 permutations under the null hypothesis that there is no difference in dispersion between groups.

Since genetic differentiation among populations can affect epigenetic variation, we tested whether epigenetic differences detected among sites were not related to genetic differentiation. Correlation between genetic and epigenetic intersite distances was assessed from Euclidean and chord distance measured, respectively, on MSAP and microsatellite loci. Similarly, genetic diversification of a clonal lineage can also occur within a given site and thus increase epigenetic variation. Nei's gene diversity, unbiased heterozygosity *H*_E_ (Nei 1978), and mean individuals’ distance to centroid were used to estimate, respectively, intrasite genetic and epigenetic diversity. Correlations between intrasite genetic and epigenetic variation were assessed. For both correlations, Pearson's correlation coefficient was performed and significance was tested with 999 permutations.

Environmental parameters (temperature [°C]; conductivity [S/cm]; dissolved oxygen [mg/L]; pH; oxidation–reduction potential) for each sampled sites were measured using the YSI 556 Multiprobe System over a short period of a week during summer 2013 to avoid temporal variation. Euclidean distances of standardized data were used to construct a dissimilarity matrix for the different sites. These parameters were used only to assess whether environmental heterogeneity exists among sites containing the five lineages because many other parameters may influence species’ ecology (habitat types, predations, disturbance patterns, substrate, etc.).

## Results

### Epigenetic diversity in natural populations

Genomic screening of DNA methylation of the five lineages provided a total of 62 polymorphic loci from 113 reproducible fragments. Less than 3% of nonreproducible bands were removed from the analyzed data. A total of 54 loci were variable in more than one lineage, whereas eight loci were lineage specific. The number of polymorphic loci per lineage per site varied from 3 to 25 in natural populations (Table 1). The total epigenetic variation also differs among lineages, as LR‐1, ET‐1, and ET‐2 display more than twice the number of variable loci than ET‐3 and ET‐4 (Table 1). The low variability detected in some lineage/site combination is not correlated with sample size (*R* ^*2*^ **= 0.049; *P *=* *0.488).

**Figure 2:**
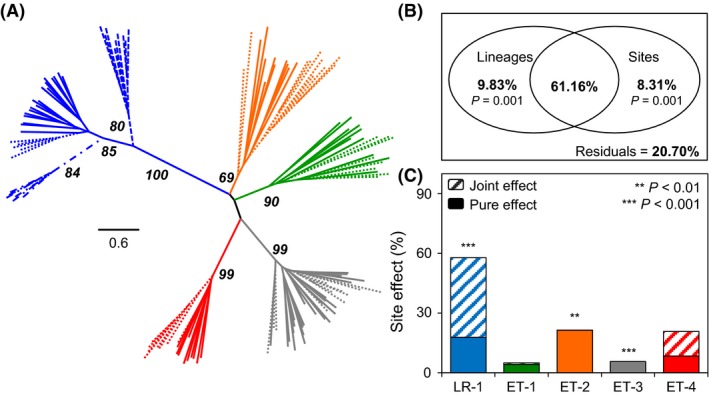
Epigenetic variation in natural populations. (A) Unrooted neighbor‐joining tree constructed from Euclidean distances between individuals’ epigenetic profile (color = lineage according to Fig. 1 legend and line type = sampled site); bold numbers refer to bootstrap support values >50% for lineages and sites. (B) Partition of the total epigenetic variation according to lineages and field sites. (C) Proportion of site effect on epigenetic variation for each lineage. Site and genetic joint effect (hatched) is separated from the pure site effect (plain) and *P‐*values refer to significance of the proportion of epigenetic variation explained uniquely by site.

Neighbor‐joining tree revealed that individuals clustered according to lineage with groups supported by very high bootstrap values for most of the lineages (Fig. 2A). Epigenetic profiles strongly vary among genotypes, even when they occur in sympatry (e.g., lineages ET‐3 and ET‐4). Individuals of the widespread lineage LR‐1 display a site‐specific epigenetic signature and clustered according to their site of capture. This sharply contrasts with lineages from the ET region where individuals of a given lineage captured in different sites are intermingled.

Partition of total epigenetic variation revealed significant contribution of lineage ($R_{a}^{2}$ = 9.83%; *P *=* *0.001) and site ($R_{a}^{2}$ = 8.31%; *P *=* *0.001). The variation explained simultaneously by both factors is much higher (61.16%; Fig. 2B) since the different lineages occur in some but not all sites. Nevertheless, results indicate that pure genetics and pure environment factors play a crucial role in epigenetic variation in *C. eos‐neogaeus*.

Partition of epigenetic variation was performed separately for each lineage to remove the impact of genetic differences. Mutations that occurred on the multiloci genotype of the clones (Table 1) were also taken into account in the partition of epigenetic variation. Results revealed that all the lineages are not plastic since only lineages LR‐1, ET‐2, and ET‐3 display a significant proportion of epigenetic changes in response to the environment (Fig. 2C). The different genotypes may display a specific epigenetic response when confronted with a given environment as observed between the sympatric lineages ET‐3 and ET‐4 (Fig. 2C). The widespread lineage LR‐1 displays the highest proportion of epigenetic variation explained by the environment ($R_{a}^{2}$ = 57.82%, 25.93% ≤ $R_{a}^{2}$ ≤ 65.96% for pairwise comparisons among sites), compared to ET lineages ($R_{a}^{2}$ ≤ 21.50%, Fig. 2C).

**Figure 3:**
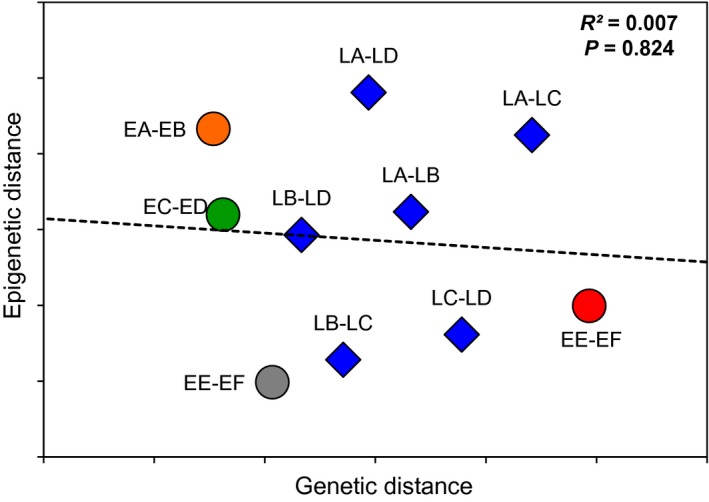
Relationship between genetic and epigenetic distances. Genetic and epigenetic distances refer, respectively, to the chord distance performed on microsatellite loci and Euclidean distance performed on methylation profiles from methylation‐sensitive amplified polymorphism analysis. Comparisons were performed among sites for each lineage. (See Fig. 1B for names and locations of sites and lineages color code.)

Comparison of sympatric lineages ET‐3 and ET‐4 revealed a high lineage effect ($R_{a}^{2}$ = 57.08%, *P *=* *0.001), indicating that great important epigenetic differences may exist among genotypes. However, the proportion of epigenetic variation explained only by intralineage genetic variation was significant for none of the lineages (*P *>* *0.05, see Table S1). Moreover, no correlation was detected between intralineage genetic and epigenetic differences among sites (*R* ^2^ = 0.007, *P *=* *0.824), indicating that greater epigenetic differences among environments are not due to higher genetic differences among populations (Fig. 3). Also, the low unexplained intrasite variation found in LR‐1 compared to ET lineages is not correlated with intrasite genetic variation (*R* ^2^ = 0.072, *P *=* *0.354). For instance, individuals of the lineage ET‐1 from the site ED are all genetically identical (*H*_E_ = 0) and display at the same time one of the highest intrasite epigenetic variation (Table 1).

**Figure 4:**
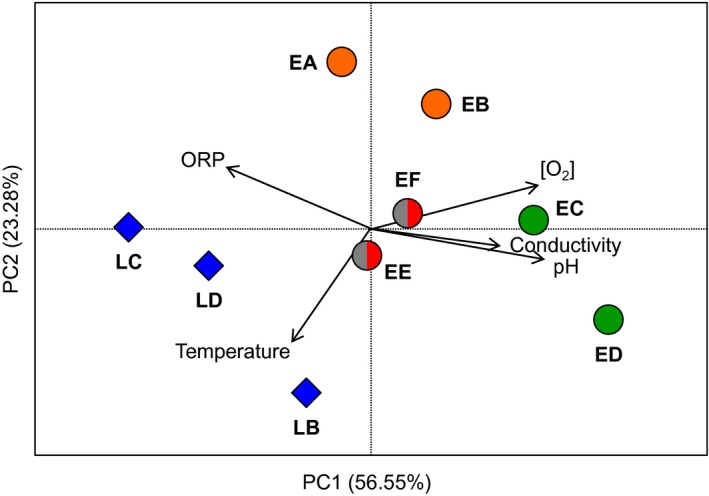
Variation of the environmental conditions in natural sites. Principal component analysis of the physicochemical parameters (temperature [°C]; conductivity [S/cm]; [O2], dissolved oxygen [mg/L]; pH; ORP, oxidation–reduction potential) among sites; lineages found in each site are represented by different colors according to Figure 1B.

Since all the different lineages of the hybrid *C. eos‐neogaeus* were not found in sympatry, they may experience different environmental variation among sites. However, no significant difference in the variation of physicochemical conditions was observed between ET and LR regions (*P *=* *0.758; Fig. 4 and Table S2).

Finally, 20.70% of the total epigenetic variation remained unexplained by lineages and environments (Fig. 2B). Unexplained variation (the variation explained neither by site environment nor microsatellite variation, Fig. 2C) accounts for a very low proportion of the total variation in the widespread lineage LR‐1, whereas it represents up to 98% of the total variation in lineages from the ET region (see Table S1).

### Common garden experiments

**Figure 5:**
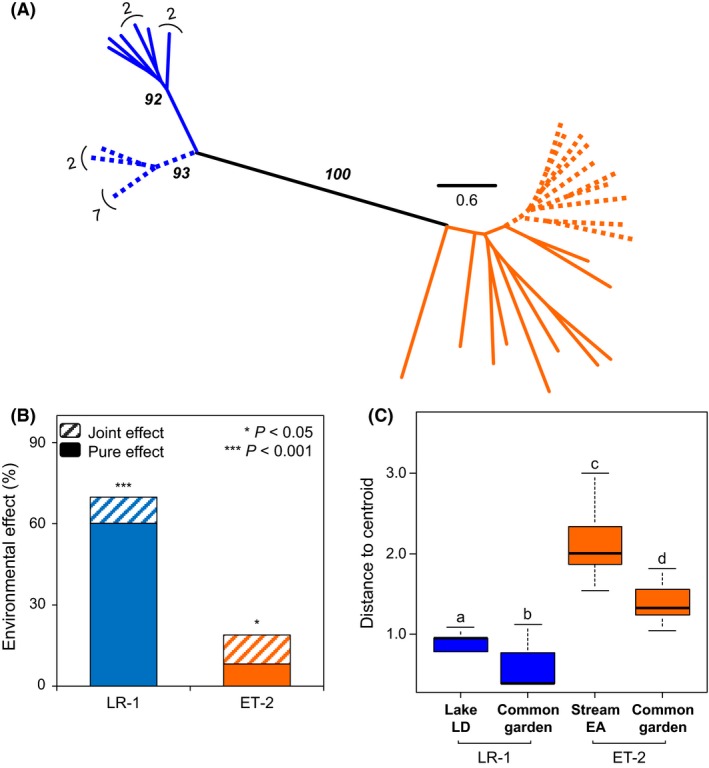
Epigenetic variation in controlled conditions. (A) Unrooted neighbor‐joining tree constructed from Euclidean distances between epigenetic profiles of individuals reared in common garden (dotted line) versus field sites (solid line). Numbers with bracket indicate the number of identical individuals and bold numbers refer to bootstrap support values >50% for lineages and sites. (B) Proportion of environmental effect on epigenetic variation. Site and genetic joint effect (hatched) is separated from the pure site effect (plain) and *P‐*values refer to significance of the proportion of epigenetic variation explained uniquely by site. (C) Boxplot with median, quartiles, and range of individuals’ distance to centroids to assess within‐environment variation. Lowercase letters refer to the results of the pairwise comparisons of group mean dispersions.

Epigenetic variability was measured on individuals reared in controlled conditions and compared to individuals from their respective sampled site for two lineages that displayed the highest epigenetic variation in natural populations (Table 1). No epigenetic variation explained uniquely by intralineage genetic variation was observed for both LR‐1 ($R_{a}^{2}$ = 0.048%, *P *=* *0.359) and ET‐2 ($R_{a}^{2}$ = 4.37%, *P *=* *0.264) lineages. For each lineage, epigenetic profiles of individuals from natural environments and those reared in controlled conditions clustered together (Fig. 5A). The different epigenetic profiles observed between lineages reared in the same controlled conditions confirm the genetic influence on the epigenetic response to the environment ($R_{a}^{2}$ = 86.57%, *P *=* *0.001).

Importantly, both LR‐1 and ET‐2 lineages responded to environmental changes as epigenetic profiles in controlled conditions differed from the ones observed in natural conditions (Fig. 5A). However, both lineages did not respond to environmental changes to the same extent. Indeed, five monomorphic loci for individuals from natural populations have changed for all individuals in controlled conditions for LR‐1, indicating a consistent response to the transfer for individuals of this lineage. At the opposite, no such loci are observed for ET‐2. This is also illustrated by the clear discrimination of LR‐1 individuals in controlled versus natural conditions supported by high bootstrap values, whereas ET‐2 individuals do not form two monophyletic groups (Fig. 5A). Partition of variation analysis confirmed that epigenetic changes associated with development in natural versus controlled conditions are more extreme for LR‐1 ($R_{a}^{2}$ = 69.83%, *P *=* *0.001) than ET‐2 ($R_{a}^{2}$ = 18.91%, *P *=* *0.001). Higher pure environmental effect is also detected for LR‐1 ($R_{a}^{2}$ = 60.14%, *P *=* *0.001) than for ET‐2 ($R_{a}^{2}$ = 8.17%, *P *=* *0.031) when taking into account the genetic variation measured with microsatellites loci. At the opposite of natural populations (Fig. 2C), only a low confounding effect with genetic variation is detected in common garden experiments (Fig. 5B). Levels of epigenetic variation measured in common gardens therefore represent a response to environmental change rather than a consequence of genetic diversity. Interestingly, epigenetic changes in response to transfer in experimental conditions are of the same magnitude as those detected between different sites in natural conditions for both lineages (LR‐1: 57.82%, ET‐2: 21.50%, Figs. 2C and 5B).

The epigenetic variation measured in individuals reared in uniform common garden conditions in the absence of mortality is expected to reveal the stochastic variation. A significant reduction in the intra‐environment variation was detected in both lineages LR‐1 (*P *=* *0.014) and ET‐2 (*P *=* *0.001), indicating that natural sites were heterogeneous compared to controlled condition (Fig. 5C). Nevertheless, the variation in ET‐2 remains significantly higher than the one detected in LR‐1 in the same environment (*P *=* *0.001; Fig. 5C). Among the monomorphic loci for individuals from the field sites, 4 of them turned to be variable in common gardens for ET‐2, whereas no such response was observed for LR‐1. Similarly, a higher proportion of epigenetic variation remains unexplained by microsatellite and environmental effects for the lineage ET‐2 (76.72%) compared to the lineage LR‐1 (29.69%), confirming the higher level of stochastic variation in ET‐2.

## Discussion

This study shows that the contribution of environmentally induced and stochastic epigenetic changes strongly differs between predictable and unpredictable environments. Results from both natural sites and common garden experiments revealed the predominant role of the environment in modulating epigenetic variation in the lineage LR‐1, as expected from stable environments (Angers et al. 2010; Pujadas and Feinberg 2012; Scheiner 2014). This lineage displayed very low amount of epigenetic changes within site, the highest proportion of epigenetic variation among natural sites, and the highest level of epigenetic changes following the transfer to experimental conditions. Significant site effect on epigenetic variation indicates that the environmental signal(s) of a site is/are consistently interpreted by individuals of the LR‐1 lineage.

On the other hand, the multiple lineages found in unpredictable environmental conditions are all characterized by high amount of epigenetic variation, but more particularly by a high contribution of stochastic epimutations. The high level of stochastic variation in the lineage ET‐2 is well illustrated by several monomorphic loci in natural populations that turned out to be polymorphic among individuals reared in a homogenous environment, whereas no such loci were observed for the lineage LR‐1 from stable environments. Several processes resulting in diverse error rates are responsible for stochastic establishment of epigenetic marks (Bird 2002). Even if the mechanistic details behind these processes are not completely understood yet, variation in the perception and/or interpretation (Botero et al. 2015) of environmental cues can, for instance, result in some errors/variations that may explain epigenetic variation among individuals.

Altogether, our results suggest that organisms found in predictable environments display environmentally induced epigenetic changes, whereas those living in unpredictable environments mostly exhibit randomly established epigenetic marks. This relationship between the level of environmentally induced epigenetic variation and the predictability of environmental conditions is consistent with our predictions.

### Epigenetics, genetics, and environment

The epigenetic changes observed in all individuals transferred from natural to experimental conditions confirm the environmental influence on epigenetic variation and highlight the rapid epigenetic response of individuals following changes in environmental conditions. Epigenetic variation measured within natural populations may result from stochastic epigenetic variation but also from plasticity due to the heterogeneity of environmental conditions within a given site. The reduction in within‐site variation observed in controlled conditions suggests that environmental heterogeneity may account for a part of the observed epigenetic variation within natural environments. Such a reduction in variation could not be attributed to the elimination of unfit phenotypes resulting from random changes because no mortality was recorded during the experiment. The epigenetic variation detected within a given lineage in common garden experiments is therefore expected to represent spontaneous stochastic epigenetic modifications. As expected, stochastic variation accounts for the lowest amount and the lowest proportion of the total variation in the lineage LR‐1.

The epigenetic response to a given environmental signal is, however, strongly influenced by the genotype as evidenced by the distinct sympatric lineages in natural conditions or reared in similar experimental conditions. Because the extent of plasticity has a genetic basis (Scheiner 1993; de Jong 1995; Scheiner and Berrigan 1998), each genotype may have a different perception or interpretation of environmental signals, resulting in different environmentally induced epigenetic responses. For instance, the widespread lineage LR‐1 found in a stable and predictable system of lakes and ponds displayed higher environmentally induced epigenetic variation than ET lineages. LR‐1 may have had therefore the highest fitness in predictable environments because of its capacity to adjust its phenotype to a wide range of environmental conditions, explaining the higher success of colonization of this lineage in the LR region (Angers and Schlosser 2007).

Genomic composition is known to play an important role for obligatory and/or facilitated epigenetic variation (Morgan et al. 1999; Richards 2006; Lippman and Zamir 2007; Furrow and Feldman 2014). Because of this genetic effect, accumulation of mutations with coalescent time within a lineage could make individuals of a sublineage more epigenetically similar than individuals from different sublineages. Long‐term inheritance of epigenetic variation (Bossdorf et al. 2008; Jablonka and Raz 2009; Bollati and Baccarelli 2010) may also lead to the very same results. However, these factors did not explain the epigenetic variation measured within lineages as no significant pure genetic effect was detected. Comparison of individuals from the same population but reared in different environments (field sites vs. controlled conditions) confirmed the lack of correlation between genetic and epigenetic variation within a given lineage as no genetic effect was detected either. Moreover, the high environmental and genetic joint effect observed among natural populations was strongly reduced in controlled conditions, while the total environmental effect remained to the same extent as the one measured in natural conditions. This is in accordance with a previous study where individuals presenting the same genotype but found in different environments were epigenetically more different than distinct genotypes found within the same environment (Massicotte and Angers 2012).

The lack of correlation between pairwise genetic and epigenetic distances indicates that higher epigenetic differences among sites for the lineage LR‐1 cannot be attributed to higher genetic differences among LR sites. Similarly, the higher unexplained variation in ET lineages could not be attributed to higher genotypic diversity. Furthermore, using physicochemical parameters as a surrogate to environmental conditions, sites from the LR region are as different as sites from the ET region. Therefore, the largest environmentally induced epigenetic variation detected in the LR‐1 lineage could not be attributed to a larger environmental difference among sites in this region.

### Epigenetics, plasticity, and bet‐hedging

Phenotypic plasticity and diversified bet‐hedging are two ecological models developed to describe how organisms maximize their fitness in changing environments (Slatkin 1974; Stearns 1989; DeWitt et al. 1998; Simons 2011). However, both strategies rely essentially on phenotypic variability in coping with environmental fluctuations, explaining why different sources of epigenetic variation have been proposed as the underlying mechanisms of these strategies (Bossdorf et al. 2008; Angers et al. 2010; Piggot 2010; Casadesús and Low 2013; Herman et al. 2014; Vogt 2015). Environmentally induced epigenetic changes underline the capacity of a genotype to adjust the phenotype to match the environment for plasticity, while stochastic epigenetic changes highlight the propensity to randomly diversify the phenotypes for bet‐hedging.

The exact environmental parameters or genes associated with the survival of individuals can be different or unknown according to the studied organisms. Thus, in contrast to previous empirical studies focussing on phenotypic traits or fitness to assess the consequences of plasticity or bet‐hedging, we rather assessed the inherent mechanisms of these strategies, namely epigenetic variation, to evaluate the capacity of a genotype to be plastic or bet‐hedger. As expected, our results are fully consistent with theoretical studies, revealing that the contribution of the different sources of epigenetic variation is clearly different according to the environmental uncertainty. In addition, for a given lineage, each source of epigenetic changes is largely predominant at the level of the genome, with environmentally induced epigenetic marks for the lineage in predictable environments or random epimutations for the different lineages found in unpredictable environments. Therefore, we suggest the contribution of environmentally induced versus spontaneous stochastic epigenetic variation may provide information about the capacity of a genotype to modify its phenotype according to environmental conditions or, alternatively, to display phenotypic variation notwithstanding environmental conditions.

### Two sides of the same coin?

Plasticity and diversifying bet‐hedging models seem to be completely opposite to one another in their perception and their response to the environment. However, our results reveal that their respective source of epigenetic variation acts conjointly, while at a different extents according to environmental uncertainty. The inherent mechanisms are not alternative to each other but complementary in producing phenotypic diversity. This also indicates that plasticity and diversifying bet‐hedging are not mutually exclusive strategies.

Because variation in the predictability of environmental conditions may be encountered, compromise between plasticity and bet‐hedging strategies may be optimal. Our results indicated that even when individuals display a consistent response to environmental signals, random changes occurred resulting in stochastic variation around the optimal epigenetic profile. Such a “mixed strategy” may represent a compromise between pure strategies that can be considered as both extremes of the response to environmental uncertainty. For instance, it may provide a higher survival rate than only bet‐hedging if environment changes turned to be temporarily predictable as well as a higher survival rate than only plasticity if changes are occasionally unpredictable. This idea of maximizing sources of phenotypic variation has been already explore in the conditional bet‐hedging (Jablonka et al. 1995). This concept is based on the principle that some individuals of a population will modify their phenotypes according to environmental changes, whereas the rest of the population will retain phenotypes of the previous generation whatever the environmental conditions (Jablonka et al. 1995).

## Conclusion

This study performed in natural populations and controlled conditions provides strong empirical evidence that different sources of epigenetic variation can be conjointly used, but at different extents to cope with environmental uncertainty. Because both environmentally induced and stochastic epigenetic changes may result in phenotypic variation, they are expected to play a crucial role in plasticity and bet‐hedging strategies, although this remains to be formally demonstrated for the latter strategy. This study also highlights the importance of recognizing these distinct sources of epigenetic variation because total variation is not sufficient to assess the capacity of a genotype to respond to environmental changes. Therefore, we believe this study provides a framework to further assess the potential of organisms to respond to environmental changes.

## Conflict of Interest

None declared.

## Supporting information


**Table S1.** Partition of epigenetic variation.

**Table S2.** Physico‐chemical parameters of natural environments.

**Table S3.** Genetic and epigenetic raw data.
