# Supplementary material for: Facing environmental predictability with different sources of epigenetic variation
Source: Ecol Evol. 2016 Jun 28;6(15):5234–45. doi: 10.1002/ece3.2283 (PMC4984500; doi:10.1002/ece3.2283)
Supplement: Supplementary file 1 — Table S1. Partition of epigenetic variation. [file ECE3-6-5234-s001.docx]

**Table S1. Partition of epigenetic variation.** Details of the partition of epigenetic variation performed among environments for each lineage. The fractions explained uniquely by environment (field sites or common garden) and genetics (differences among individuals) are respectively [a] and [c], and their joint effect is [b]; [d] = residuals. Significance of the *R^2^_a_* values for [a] and [b] was tested by permutation tests using 999 randomizations. Data used for those analyses are uploaded as Supporting Information S3 Dataset.

Environment

[a]

Genetics

[c]

[b]

[d] = Residuals

| **Lineage** | **Environments** |  | **Df** | ***R^2^*** | ***R^2^_a_*** | ***P* value** |  |
| --- | --- | --- | --- | --- | --- | --- | --- |
| ET-1 | EC *vs* ED | [a+b] | 1 | 0.10597 | 0.05010 | - |  |
|  |  | [a] | 1 |  | 0.04219 | 0.094 | . |
|  |  | [b] | 0 |  | 0.00791 | - |  |
|  |  | [c] | 3 |  | -0.03756 | 0.86 |  |
|  |  | [d] |  |  | 0.98746 | - |  |
| ET-2 | EA *vs* EB | [a+b] | 1 | 0.25635 | 0.21504 | - |  |
|  |  | [a] | 1 |  | **0.21391** | **0.003** | ****** |
|  |  | [b] | 0 |  | 0.00113 | - |  |
|  |  | [c] | 8 |  | 0.02578 | 0.358 |  |
|  |  | [d] |  |  | 0.75919 | - |  |
| ET-2 | EA *vs* CG (Aquariums) | [a+b] | 1 | 0.23177 | 0.18909 | - |  |
|  |  | [a] | 1 |  | **0.08170** | **0.031** | ***** |
|  |  | [b] | 0 |  | 0.10740 | - |  |
|  |  | [c] | 7 |  | 0.04372 | 0.264 |  |
|  |  | [d] |  |  | 0.76719 | - |  |
| ET-3 | EE *vs* EF | [a+b] | 1 | 0.07352 | 0.05036 | - |  |
|  |  | [a] | 1 |  | **0.05686** | **0.001** | ******* |
|  |  | [b] | 0 |  | -0.00651 | - |  |
|  |  | [c] | 6 |  | 0.04074 | 0.08 | . |
|  |  | [d] |  |  | 0.90890 | - |  |
| ET-4 | EE *vs* EF | [a+b] | 1 | 0.25416 | 0.20755 | - |  |
|  |  | [a] | 1 |  | 0.08417 | 0.063 | . |
|  |  | [b] | 0 |  | 0.12337 | - |  |
|  |  | [c] | 6 |  | 0.11148 | 0.055 | . |
|  |  | [d] |  |  | 0.68098 | - |  |
| LR-1 | LA, LB, LC, LD | [a+b] | 3 | 0.60035 | 0.57815 | - |  |
|  |  | [a] | 3 |  | **0.17809** | **0.001** | ******* |
|  |  | [b] | 0 |  | 0.40006 | - |  |
|  |  | [c] | 7 |  | -0.01434 | 0.936 |  |
|  |  | [d] |  |  | 0.43619 | - |  |
| LR-1 | LD *vs* CG (Aquariums) | [a+b] | 1 | 0.71603 | 0.69828 | - |  |
|  |  | [a] | 1 |  | **0.60142** | **0.001** | ******* |
|  |  | [b] | 0 |  | 0.09686 | - |  |
|  |  | [c] | 2 |  | 0.00481 | 0.359 |  |
|  |  | [d] |  |  | 0.29691 | - |  |
| LR-1 | LA *vs* LB | [a+b] | 1 | 0.42497 | 0.40983 | - |  |
|  |  | [a] | 1 |  | 0.00079 | 0.375 |  |
|  |  | [b] | 0 |  | 0.40904 | - |  |
|  |  | [c] | 2 |  | -0.00792 | 0.780 |  |
|  |  | [d] |  |  | 0.59809 | - |  |
| LR-1 | LA *vs* LC | [a+b] | 1 | 0.65952 | 0.64061 | - |  |
|  |  | [a] | 1 |  | 0.00031 | 0.461 |  |
|  |  | [b] | 0 |  | 0.6403 | - |  |
|  |  | [c] | 4 |  | -0.03976 | 0.956 |  |
|  |  | [d] |  |  | 0.39915 | - |  |
| LR-1 | LA *vs* LD | [a+b] | 1 | 0.67966 | 0.65964 | - |  |
|  |  | [a] | 1 |  | **0.18636** | **0.001** | ******* |
|  |  | [b] | 0 |  | 0.47328 | - |  |
|  |  | [c] | 2 |  | -0.00801 | 0.641 |  |
|  |  | [d] |  |  | 0.34837 | - |  |
| LR-1 | LB *vs* LC | [a+b] | 1 | 0.27826 | 0.25927 | - |  |
|  |  | [a] | 1 |  | **0.04422** | **0.012** | ***** |
|  |  | [b] | 0 |  | 0.21505 | - |  |
|  |  | [c] | 6 |  | -0.01522 | 0.707 |  |
|  |  | [d] |  |  | 0.75595 | - |  |
| LR-1 | LB *vs* LD | [a+b] | 1 | 0.46208 | 0.44714 | - |  |
|  |  | [a] | 1 |  | **0.03811** | **0.004** | ****** |
|  |  | [b] | 0 |  | 0.40902 | - |  |
|  |  | [c] | 2 |  | 0.00371 | 0.34 |  |
|  |  | [d] |  |  | 0.54916 | - |  |
| LR-1 | LC *vs* LD | [a+b] | 1 | 0.59392 | 0.56854 | - |  |
|  |  | [a] | 1 |  | **0.08731** | **0.002** | ****** |
|  |  | [b] | 0 |  | 0.48123 | - |  |
|  |  | [c] | 4 |  | 0.03347 | 0.138 |  |
|  |  | [d] |  |  | 0.39799 | - |  |
