# Supplementary material for: Facing environmental predictability with different sources of epigenetic variation
Source: Ecol Evol. 2016 Jun 28;6(15):5234–45. doi: 10.1002/ece3.2283 (PMC4984500; doi:10.1002/ece3.2283)
Supplement: Supplementary file 2 — Table S2. Physico‐chemical parameters of natural environments. [file ECE3-6-5234-s002.docx]

**Table S2. Physico-chemical parameters of natural environments.** Site codes refer to sites in Fig 1.

| **Sites** | **Temperature (°C)** | **Conductivity (S/cm)** | **Disolved oxygene (mg/L)** | **pH** | **Oxidation-reduction potential** |
| --- | --- | --- | --- | --- | --- |
| LB | 29.06 | 48 | 5.69 | 7.83 | 27.60 |
| LD | 23.37 | 52 | 3.66 | 7.29 | 48.6 |
| LC | 23.40 | 64 | 3.08 | 6.61 | 59.00 |
|  |  |  |  |  |  |
| EA | 12.46 | 43 | 9.21 | 7.38 | 49.02 |
| EB | 13.01 | 82 | 10.28 | 8.02 | 40.10 |
| EC | 16.31 | 384 | 9.43 | 8.04 | 27.6 |
| ED | 20.70 | 230 | 11.30 | 8.95 | 9.30 |
| EE | 16.84 | 138 | 5.02 | 7.33 | 15.20 |
| EF | 17.68 | 33 | 8.55 | 8.02 | 26.90 |
